# The role of polygenic risk score gene-set analysis in the context of the omnigenic model of schizophrenia

**DOI:** 10.1038/s41386-019-0410-z

**Published:** 2019-05-11

**Authors:** Alexandros Rammos, Lara A. Neira Gonzalez, The Schizophrenia Working Group of the Psychiatric Genomics Consortium 2,, Daniel R. Weinberger, Kevin J. Mitchell, Kristin K. Nicodemus

**Affiliations:** 1 Smurfit Institute of Genetics and Institute of Neuroscience, Trinity College Dublin, Dublin, Ireland; 2 Centre for Genomic and Experimental Medicine, Institute of Genetics and Molecular Medicine, University of Edinburgh, Edinburgh, UK; 3 Lieber Institute for Brain Development, Johns Hopkins Medical Campus, Baltimore, MD USA; 4 Centre for Cognitive Ageing and Cognitive Epidemiology, University of Edinburgh, Edinburgh, UK

**Keywords:** Schizophrenia, Genetics

## Abstract

A recent development in the genetic architecture of schizophrenia suggested that an omnigenic model may underlie the risk for this disorder. The aim of our study was to use polygenic profile scoring to quantitatively assess whether a number of experimentally derived sets would contribute to the disorder above and beyond the omnigenic effect. Using the PGC2 secondary analysis schizophrenia case-control cohort (*N* = 29,125 cases and 34,836 controls), a robust polygenic signal was observed from gene sets based on TCF4, FMR1, upregulation from MIR137 and downregulation from CHD8. Additional analyses revealed a constant floor effect in the amount of variance explained, consistent with the omnigenic model. Thus, we report that putative core gene sets showed a significant effect above and beyond the floor effect that might be linked with the underlying omnigenic background. In addition, we demonstrate a method to quantify the contribution of specific gene sets within the omnigenic context.

## Introduction

Schizophrenia is a highly heritable disorder showing complex genomic architecture. Genome-wide association studies (GWASs) have been used to identify the common variants contributing to the risk of disease and measure their collective effect. A recent schizophrenia GWAS [1] identified over a hundred common single-nucleotide polymorphisms (SNPs) at genome-wide significance levels, though the effect of each SNP on its own was modest.

The underpinning architecture of schizophrenia remains unclear [2]. Several methods have been applied to capture cumulative common variation that might confer vulnerability, including polygenic risk scores (PRSs) [3, 4]. PRSs use the coefficients derived from a discovery GWAS as weights for each SNP allele in order to calculate an overall risk score for each individual in an independent sample. PRSs are capable of explaining some proportion of overall variance in liability.

In order to identify the underlying biological pathways, gene-set enrichment analyses have been conducted using categories defined by gene ontology or by biochemical interaction with the products of high-risk genes [5]. Methods such as MAGMA [6], INRICH [7] and ALIGATOR [8] have allowed the in-depth exploration of GWAS results in terms of finding biochemical pathway enrichment, and have been crucial in expanding our understanding of potential underlying mechanisms of complex traits. However, despite their prominence in GWAS [9, 10] and exome-sequencing studies [11], these analyses do not estimate the contribution of these gene sets to the amount of variance explained; instead, they state whether the gene set is more enriched, in terms of GWAS *p*-values, than expected by chance.

A recent paper [12] suggested that SNPs in all genes expressed in the relevant tissue (e.g., brain in schizophrenia) make a contribution to heritability and polygenic risk. Within that context there are two types of genes, *core* and *peripheral*, that confer risk. Genes identified in GWASs or rare-variant studies may be core genes that serve as the basis of developing networks used to identify peripheral genes.

Our study aimed to quantitatively assess whether specific gene sets, centred on putative core genes, make a larger-than-expected contribution to polygenic risk. We focused on eight gene sets, six of which are centred on genes previously implicated in schizophrenia risk. We hypothesized that these sets would be associated with schizophrenia case-control status at a greater-than-expected level. The remaining two gene sets were associated with cancer and cardiac disease (CD). The rationale behind the choice of each included gene set is presented below. For comparison, we examined the behaviour of the PRSs under H_0_. Finally, to investigate the omnigenic hypothesis on schizophrenia risk [12], we generated linkage disequilibrium (LD) independent random genic and non-genic SNP sets of equal size to the gene sets investigated. Comparison of these sets to the putative core gene sets may produce a better estimate of their contribution under the omnigenic model.

The six schizophrenia core gene-associated (SCGA) target gene sets were selected from recent studies [13–16] based on transcriptional or molecular interactions with schizophrenia putative core genes. SNPs in the gene transcription factor 4 (TCF4) are genome-wide significantly associated with risk for schizophrenia [1, 17], and haploinsufficiency of this gene causes Pitt–Hopkins syndrome, associated with severe cognitive deficits [18, 19] and risk for psychosis [20]. The TCF4 gene set was created on the basis of the differential expression of genes in neuroblastoma cells after knockdown of TCF4 [13]. A total of 1052 autosomal genes (5652 SNPs) demonstrating differential expression were included in the gene set. FMR1 (Fragile X metal retardation 1) is a gene coding for FMRP (fragile X mental retardation protein), whose loss of function results in fragile X syndrome [21], often co-morbid with autism spectrum disorders. FMR1 mutations have been linked with cognitive impairment and earlier age of onset in schizophrenia [22]. The FMRP gene set was created on the basis of functional gene sets based on developmental expression of genes contingent on FMRP expression [14]. All four gene subsets were combined into one gene set containing 680 autosomal genes (5833 SNPs). MIR137 is a microRNA with high levels of expression in the brain and neural stem cells [23]. Transcriptional targets of MIR137, such as ZNF804A and *CACNA1C*, as well as the gene itself, have been implicated with schizophrenia [17, 24, 25]. The third and fourth gene sets were chosen on the basis of the work of Hill et al. [15], where two gene sets were generated from upregulated (817 genes and 7796 SNPs) and downregulated (761 genes and 8533 SNPs) genes after overexpression of MIR137 in neural progenitor cells in vitro. *CHD8* (Chromodomain Helicase DNA Binding Protein 8) codes for a DNA helicase that suppresses gene expression by affecting chromatin restructure, and is a significant contributor to autism susceptibility [26] and CHARGE syndrome (a congenital deaf–blindness syndrome) through its interaction with CHD7 [27]. Rare variants in CHD8 may contribute to schizophrenia risk [28]. The final neural gene sets were generated from the findings of Sugathan et al. [16], where CHD8 reduction in neural progenitor cells led to the creation of two gene sets, one of upregulated (1140 genes and 8807 SNPs) and the other of downregulated (616 genes and 4986 SNPs) genes. For the latter two gene sets, the decision to split them into downregulated and upregulated gene sets was based on reports [15, 16] describing a more pronounced response under one of the conditions. Additionally, we selected two gene sets that were related to CD and cancer, drawn from the CD database (http://www.bioguo.org/CADgene/) and the Atlas of Genetics and Cytogenetics in Oncology and Haematology (atlasgeneticsoncology.org). Those gene sets had 534 and 459 genes, respectively (with 8078 and 7316 SNPs). The rationale for using these non-schizophrenia gene sets was mainly to serve as null sets of roughly equal size to the SCGA gene sets.

## Materials and methods

### The Schizophrenia Working Group of the Psychiatric Genomics Consortium 2 case-control GWASs

Sample composition and selection is described in detail in Ripke et al. [1]. In brief, cases were selected based on a diagnosis of either schizophrenia or schizoaffective disorder, as the two disorders tend to aggregate together in family studies [29] and there is a low inter-rater reliability across the two groups on the basis of their initial diagnosis [30]. The quality of diagnosis for cases was assessed through a questionnaire examining quality control and diagnosis procedures [1]. Studies with different case ascertainment procedures were included in the final sample [31]. Two of the studies included cases that were selected on the basis of clozapine prescription and a prior diagnosis of treatment-resistant schizophrenia [32]. In total, 39 different studies were included. The sample was composed of 29,125 cases and 34,836 controls of European ancestry. There were 36,318 males, 22,061 females and 5582 participants with no sex information. Details of subject composition for each individual study and how these were collected, as well as details about ethics committee review and written informed consent, can be found in Ripke et al. [1] and in the appendix (Supplementary Table S1). Genotypes were imputed using the 1000 Genomes Project dataset (August 2012, 30,069,288 variants, release “v3.macGT1”) as a reference for the imputation process, through the use of IMPUTE2/SHAPEIT [33]. Quality control excluded the following: SNP missingness < 0.05 (before sample removal), subject missingness < 0.02, autosomal heterozygosity deviation (|Fhet| < 0.2), SNP missingness < 0.02 (after sample removal), difference in SNP missingness between cases and controls < 0.02, and SNP Hardy–Weinberg equilibrium (*p*-value > 10^−6^ in controls or *p*-value > 10^−10^ in cases).

### Leave-one-out (LOO) PRS analysis

**Fig. 1:**
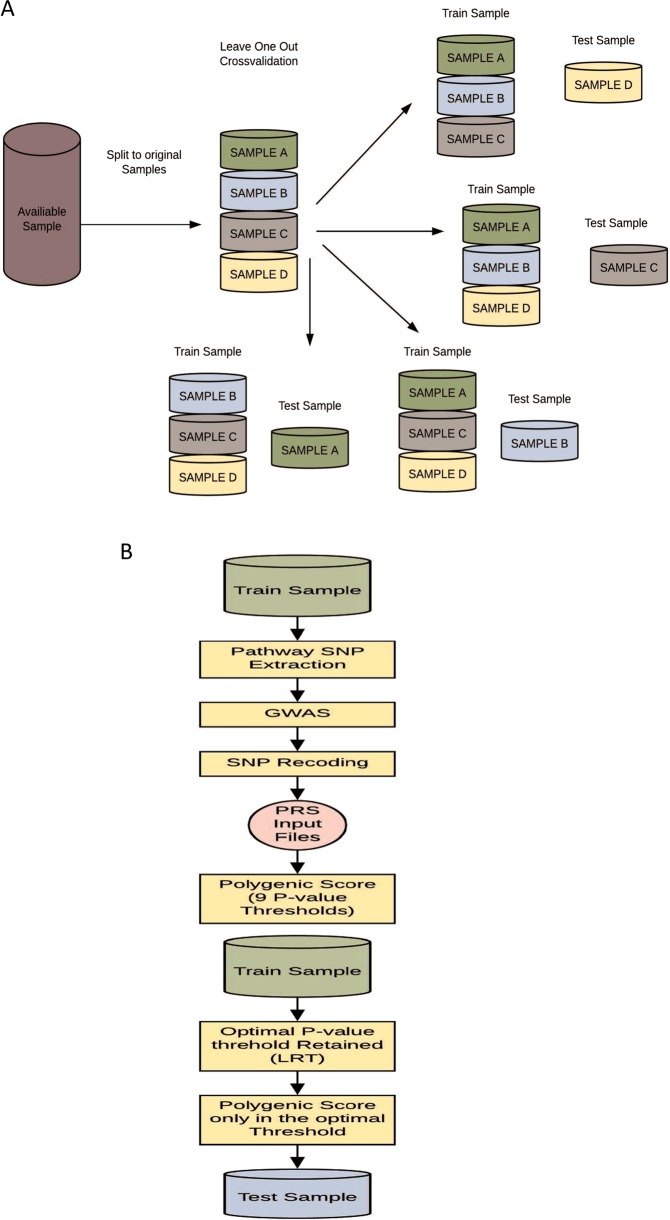
**a** Leave-one-out cross-validation process. Example of leave-one-out cross-validation process for a sample containing four datasets. The same process was followed with the 39 PGC datasets. **b** Flowchart for polygenic score generation in each leave-one-out Iteration. Flowchart of the process followed in each iteration of the leave-one-out cross-validation. PRS input files are the polygenic scoring file and the individual SNP *p*-value file. *PRS* polygenic risk score; *LRT* likelihood ratio test

Two datasets were created for each of the 39 studies: one with every dataset but the held-out set, serving as the training set; and the other with the held-out set, serving as the independent testing set. For each study, a GWAS was performed in the training set to calculate the *p*-value and ln(odds ratio) of each individual SNP (Fig. 1a). To confirm that SNPs in the training set were coding the same reference allele as the risk allele in the test study, we coded all SNPs as risk by selecting the allele with OR > 1. Afterwards, PRSs were created for nine different *p*-value cut-off thresholds (0.0001, 0.001, 0.01, 0.05, 0.10, 0.20, 0.30, 0.40 and 0.50). These were generated for the training set to reduce the need of correction for multiple testing on the held-out test set. A logistic regression model was fitted for each of these nine scores in each of the 39 training sets that included 38 studies, including covariates (count of valid genotypes, principle components and study indicators) [1]. In each study, the largest test statistic from the nine scores in the training set was used to select the single PRS to be tested on each of the 39 held-out test sets (Fig. 1a).

### Statistical analysis

All analyses were performed in PLINK 1.90 [34] for PRS generation and genetic data manipulation, and in R 3.2.4 for the generation of regression models. The R package fmsb [35] was used to calculate Nagelkerke’s *R*^2^. Note that the use of *R*^2^ here is to indicate the percentage of variation in case status explained; the use of *r*^2^ below indicates the LD or correlation between alleles at two SNPs. MetaP (Dongliang G, Duke Institute for Genome Sciences and Policy, NC, USA) was used to perform Stouffer’s *Z* *p*-value meta-analysis [36]. The gene sets described above included only the autosomal SNPs. LD pruned the SNP discovery set in PLINK using a sliding window of 50 SNPs, a sliding step of 5 SNPs and an *r*^2^ threshold of inclusion at 0.25. For the regression analysis, the original [1] principal components were used to control for population stratification, adding the study indicators as covariates. Finally, we used likelihood ratio tests between nested regression models and calculated the Nagelkerke *R*^2^ and the *p*-value for the PRS in each of the 39 held-out test datasets.

### Meta-analysis

To estimate the significance of the results in the overall sample, we performed a meta-analysis of the 39 results from the test sets only, using Stouffer’s *Z* *p*-value in metaP, also accounting for directionality of effect and sample size. Because each training set would have different ln(odds ratios) and *p*-values, each PRS was different; we thus combined *p*-values. For the Nagelkerke nested *R*^2^ values, we provided the median, interquartile range and range from the held-out test sets.

### Simulation and validation studies

We performed two additional studies to examine the methodology used and the influence of genic versus non-genic SNPs, as genic SNPs might produce inflated results [37]. The first analysis was a standard experiment-wise randomization test on the TCF4 gene set, consisting of permuting the phenotype 100 times and rerunning the entire experimental pipeline, leaving a single study out at a time, on these randomly generated phenotypes (Supplementary Fig. 1). If the pipeline is robust to type I error, 5% of these permuted experiment-wise results should show a significant result at *α* = 0.05. For the second analysis, we generated 50 random subsets of genic SNPs, defined as SNPs found within genes, 5 kb upstream of genes or 1 kb downstream of genes, of a mean size of 5000 SNPs and an equal number of non-genic SNP subsets, defined as SNPs not included in the genic subset. All SNPs were pruned at an *R*^2^ cutoff of 0.01 beforehand to make sure that only independent SNPs were selected. We ran the pipeline with all methods as previously outlined to establish if there was an omnigenic effect consistently present across the random sets and if there was a further systematic enrichment of the genic SNPs sets.

## Results

### Gene set characteristics

**Fig. 2:**
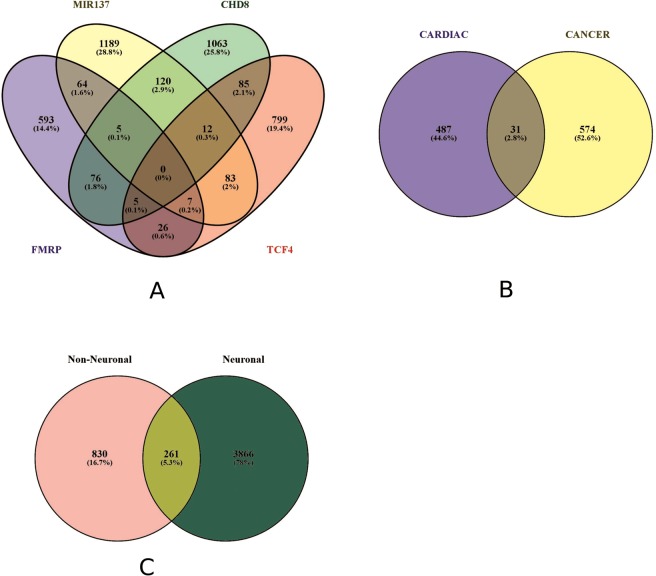
Overlap of gene sets. **a** Neuronal gene sets. **b** Non-neuronal gene sets. **c** Combination of a and b. Percentages in the graph indicate the percentage of the total genes found in each overlapping segment. MIR137 and CHD8 indicate all the genes for both the down- and the upregulated gene sets as there was no overlap between the two

Initially, we investigated if there was any overlap among the SCGA and control gene sets. We found little overlap among any gene sets (Fig. 2). In the SCGA gene sets, there were no overlapping genes in all four sets, and no two sets overlapped by more than 3% of the total genes shared. The CD and cancer gene sets showed minimal overlap between them (31 genes, 2.8%). Finally, the SCGA and non-SCGA sets had an overlap of 261 genes (5.3%). The biggest groups among overlapping genes were protein-binding genes, signalling molecule genes and receptor molecule genes.

### PRS analysis

**Fig. 3:**
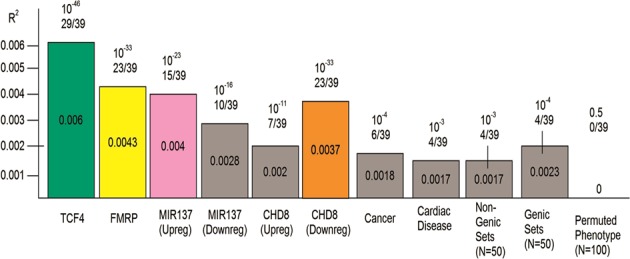
*R*^2^ and *p*-values from meta-analysis of all gene sets. Numbers on top of the bars denote the meta-analysed Stouffer’s *Z* *p*-value for the gene set and the number of polygenic scores that were significant in independent, held-out test studies. For the genic and non-genic sets, the statistics represent the median of 50 sets; the line above the box represents the range of these sets for the 50 iterations of each. The final box is the median results for 100 permuted phenotype iterations

TCF4 gene-set-weighted scores were the most strongly associated in the meta-analysis (Stouffer’s *Z* *p*-value = 1.18 × 10^−46^; Fig. 2). This particular gene set was one where most of the individual studies, as independent test sets in the LOO, were significant (29/39), showing evidence for association at *p*-value < 0.05 (uncorrected, as only one score was tested in each of the held-out test sets; Table 1). This gene set explained the highest percentage of variability among the studies described (Nagelkerke *R*^2^ = 0.6%; Fig. 3). In the original PGC2 study [1], TCF4 was GWAS-significantly associated with schizophrenia, and thus might have been driving the results. To test this, 12 SNPs within TCF4 were removed and the analysis was repeated, with results at the same level of significance (Stouffer’s *Z* *p*-value = 4.28 × 10^−40^) and effect size (Nagelkerke *R*^2^ = 0.6%). FMRP gene-set-weighted scores were also significant (Stouffer’s *Z* *p*-value = 1.66 × 10^−33^), with 23/39 individual independent test set results showing evidence for association; it explained 0.43% of the schizophrenia case-control status. For the two MIR137-regulated gene sets, we observed significant association with schizophrenia with Stouffer’s *Z* *p*-value = 3.28 × 10^−23^ for the upregulated gene set, and Stouffer’s *Z* *p*-value = 1.06 × 10^−11^ for the downregulated gene set, explaining 0.4% and 0.28%, respectively, of schizophrenia case status. For the CHD8 gene set PRSs, the downregulated gene set was significant (Stouffer’s *Z* *p*-value = 1.91 × 10^−33^) and explained 0.37% of the variability. The scores created from the upregulated genes were also significant (Stouffer’s *Z* *p*-value = 1.73 × 10^−11^), but only a small number of individual held-out test sets were individually significant (7/39) and the overall effect explained 0.2% of the variability.

**Table 1:** Nested *R*^2^ results for all individual studies for each gene set

Study details			Gene sets							
Study ID	Case (*N*)	Control (*N*)	*TCF4*	FMRP	*MIR137* (up)	*MIR137* (down)	*CHD8* (up)	*CHD8* (down)	Cancer	Heart disease
clm2	3426	4085	**0.00400**	**0.00132**	**0.00266**	0.00034	0.00046	0.00000	**0.00470**	0.00130
mgs2	2638	2482	**0.00840**	**0.00741**	**0.00534**	**0.00596**	**0.00469**	**0.00415**	**0.00401**	**0.00515**
clo3	2105	1975	**0.01693**	**0.01226**	0.01090	0.01455	0.01039	**0.04791**	0.01627	0.00143
s234	1980	2274	**0.00584**	**0.00232**	**0.00207**	0.00084	**0.00258**	**0.00113**	0.00053	**0.00179**
swe5	1764	2581	**0.00636**	**0.00761**	**0.00618**	**0.00397**	**0.00498**	**0.00379**	**0.00333**	**0.00325**
irwt	1291	1006	**0.00980**	**0.01104**	**0.00764**	0.00005	0.00312	**0.01148**	**0.01386**	**0.00609**
gras	1067	1169	**0.00820**	**0.00433**	**0.00765**	**0.00230**	0.00407	**0.00965**	0.00044	0.00246
swe6	975	1145	**0.00970**	**0.00366**	**0.02518**	0.00414	0.00202	**0.00305**	0.00175	0.00221
ajsz	894	1594	**0.00434**	**0.00592**	0.00172	**0.00850**	0.00249	**0.00154**	0.00227	0.00001
aber	719	697	**0.00623**	**0.00832**	0.00314	0.00093	0.00171	**0.01189**	0.00026	0.00070
ucla	700	607	**0.00835**	**0.00573**	0.00251	0.00266	0.00005	0.00174	0.00007	0.00327
uktr	649	649	0.00911	0.00047	0.03779	0.03675	0.00208	**0.00358**	0.00690	0.00031
pewb	574	1812	**0.00603**	**0.00211**	**0.00317**	0.00158	0.00100	**0.00565**	**0.00686**	0.00108
cou3	530	678	**0.00725**	0.00817	**0.01952**	0.00010	0.00551	**0.00599**	0.00131	0.00455
lemu	516	516	0.00011	0.00023	0.00017	0.00935	0.00013	0.00001	0.00182	0.00425
uclo	509	485	**0.00528**	**0.01287**	0.00618	**0.01092**	0.00459	0.00105	0.00000	0.00158
lie5	497	389	**0.00912**	0.00144	0.00028	0.00018	0.00125	**0.00495**	0.00101	0.00638
denm	471	456	0.00068	**0.01327**	0.00062	0.00025	0.00001	0.00010	0.00220	0.00312
asrb	456	287	**0.00402**	0.00187	0.00745	0.00503	0.00061	0.00010	0.00183	0.00249
munc	421	312	**0.00558**	**0.01080**	0.00006	**0.00737**	0.00234	0.00158	0.00397	0.00134
cati	397	203	**0.01392**	**0.01451**	**0.02506**	0.00855	0.00108	**0.02120**	0.00005	0.00137
caws	396	284	0.00268	**0.00539**	0.00722	0.00231	0.00011	**0.00994**	0.00153	0.00613
top8	377	403	**0.00772**	**0.01392**	**0.00601**	0.00016	0.00024	**0.00394**	0.00003	0.00363
edin	367	284	**0.00528**	**0.04107**	0.00202	**0.01422**	0.00085	**0.01435**	0.00378	0.00280
port	346	215	0.00016	0.00135	0.00185	0.00487	0.00000	**0.00586**	0.00038	0.00021
umeb	341	577	**0.00684**	**0.00864**	0.00409	**0.01769**	**0.00659**	**0.00752**	0.00195	0.00122
msaf	325	139	0.00026	0.00064	0.00169	0.00080	0.00009	**0.00335**	0.00158	0.00009
ersw	265	319	**0.00635**	**0.00846**	0.00327	0.00351	0.00085	0.00003	0.00015	0.00675
dubl	264	839	**0.00921**	0.00025	**0.00434**	0.00224	0.00141	**0.01166**	0.02123	**0.00845**
egcu	234	1152	**0.00291**	0.00213	0.00041	0.00047	**0.00449**	0.00037	**0.00520**	0.00027
swe1	215	210	0.00133	0.00027	0.00237	0.00326	0.00221	**0.03613**	0.00243	0.01048
buls	195	608	**0.00579**	**0.00950**	**0.01389**	0.00028	**0.00979**	0.00280	0.00821	0.00119
umes	193	704	**0.01759**	0.00096	0.00041	0.00078	0.00084	0.00155	0.00026	0.00156
zhh1	190	190	0.00023	0.00111	**0.01705**	0.00005	0.00063	0.00041	0.00031	0.00195
lacw	157	245	**0.02095**	**0.02732**	0.00884	**0.01177**	**0.02024**	**0.01505**	0.00536	0.00354
pews	150	236	0.00076	0.00004	0.00038	**0.01161**	0.00008	0.00192	0.00126	0.00095
lie2	133	269	**0.00948**	0.00167	**0.01504**	0.00286	0.01548	0.00407	0.00096	0.00015
butr	70	70	**0.00577**	0.00397	0.00210	0.00075	0.00495	0.00218	0.00251	0.00005
cims	67	65	0.00005	0.00006	0.00602	0.00830	0.02008	0.00455	0.00226	0.00164

Table of results in each individual study; the first column indicates the PGC2 label used for each study. The table is sorted by the number of cases. Highlighted boxes had a level of significance *p* < 0.05. Further details for each study and their respective size can also be found in the supplement and the original PGC2 study [1]

Gene sets created from the non-SCGA sources were weakly, but still statistically significantly, associated with the outcome (Stouffer’s *Z* *p*-value = 2.14 × 10^−4^ and 1.67 × 10^−3^, respectively). Six and four out of the 39 polygenic scores were significant at *p*-value < 0.05 (uncorrected) in those analyses, respectively. To examine the distribution of *p*-values across all of the gene sets investigated, we created *p*-value bins corresponding to deciles under H_0_, where *p*-values are distributed ~*U* (0,1). For all the gene sets investigated, there was an increased proportion of SNPs in the top 10% bin for all gene sets, consistent with the quantile–quantile (Q–Q) plot from the PGC2 mega-analysis [1] and our own Q–Q plot demonstrating a deviation of the distribution of *p*-values from expected under H_0_ (Supplementary Fig. 2).

A genome-wide PRS was generated (3,848,785 SNPs) with a median nested Nagelkerke *R*^2^ value of 0.24. If each SNP contributed equally to the score, then the results for the pathways of interest should only be able to explain roughly between 0.03% (0.24/3848785*4986, for CHD8 downregulated) and 0.055% (0.24/3848785*8807, for CHD8 upregulated). It is important to note that these results were for the 39 studies without pruning, and therefore not directly comparable to the ones that were generated with the LOO process.

### Simulation and validation studies

In the simulation study that was performed, for 100 runs with permuted phenotypes, the type I error rate at *α* = 0.05 was as expected under H_0_, with 4 out of 100 having a Stouffer’s *Z* *p*-value value of less than 0.05 (i.e. type I error of 4%). In the examination of the omnigenic effect and the possible additional effect attributable to genic SNPs, 50 random subsets of 5000 independent (with LD < 0.25) genic and non-genic SNPs were generated, and the same analytical protocol described previously was implemented. On average, all sets of SNPs that were tested had a level of significance ranging from 10^−2^ to 10^−7^, with no individual set exceeding the significance of the SCGA sets. Genic sets were consistently, but only slightly, more significant than non-genic sets (median Stouffer’s *Z* *p*-value = 1.06 × 10^−4^ versus 2.54 × 10^−3^). The nested Nagelkerke *R*^2^ values were also higher in the genic set with a median value of 0.0021 versus 0.0016 for the non-genic set.

We examined our SCGA gene sets for enrichment in genes specifically expressed in nervous system tissues or for broadly expressed genes. The omnigenic model suggests that there will be enrichment in gene sets associated with schizophrenia. Results from that analysis (Supplementary Table S2) indicate an enrichment of broad terms for most of the gene sets under examination, with the exception of the FMRP gene set, which showed an excess enrichment for neuron-specific functions and more specifically nervous system development (*p*-value = 3.36 × 10^−60^) and generation of neurons (*p*-value = 6.97 × 10^−44^). For the non-SCGA gene sets, there was an enrichment for DNA regulation elements (cancer) and response to stressors (CD).

## Discussion

PRSs were used to investigate whether potential core gene sets played a significant role in the omnigenic model of schizophrenia. There was significant heterogeneity among the gene sets, with the TCF4 gene set, the FMRP gene set, the gene set upregulated in the presence of excess MIR137 and the gene set downregulated in the absence of CHD8 shown to be associated with schizophrenia. In contrast, the apparently significant effects that were observed in the control gene sets (cancer and CD), as well as the gene set downregulated in the presence of excess MIR137 and the gene set upregulated in the absence of CHD8, were not higher than a floor effect observed with random sets of genic SNPs and non-genic SNPs and could be attributable to an omnigenic or highly polygenic background [12]. Our results were not driven by gene sizes within each gene set (Supplementary Table S3).

Among the investigated sets, the TCF4 gene set was the most strongly associated with schizophrenia, with a Stouffer’s *Z* *p*-value of 1.18 × 10^−46^. The nested *R*^2^ effect observed was three times that of any set of random SNPs of the same size. The result retained its significance and magnitude of effect size even after removing SNPs within the core gene TCF4, indicating that the observed relationship exists between peripheral genes of the gene set and the phenotype above the effect that TCF4 might exert as a core gene. There is consistent evidence for the role of TCF4 in schizophrenia [1, 17]. Additionally, due to the nature of SNPs implicated (non-coding genetic elements), the pathway of genes influenced by TCF4 expression [38] might also be potentially involved in the common polygenic background of the disorder.

The FMRP gene set was also significantly associated with schizophrenia. FMRP has primarily been implicated in autism spectrum disorders. There are commonalities among both the clinical features and genomics of major psychiatric disorders and a recent cross-disorder mega-analysis GWAS [39] that indicated that common variation predisposing to mental illnesses might be shared to some degree among major psychiatric disorders. Additional evidence of the involvement of FMRP targets to schizophrenia can be observed from rare variant studies that have consistently implicated FMRP pathways with schizophrenia [40–42].

In the two MIR137 gene sets, there was a positive effect only on the gene set that was upregulated after MIR137 over expression. The downregulated gene set, although statistically significant, did not show an effect stronger than what would be expected by the omnigenic model using randomly selected genic SNPs. This result is consistent with findings of other studies of MIR137 expression indicating that upregulation of the gene is linked with pathways implicated in psychosis (such as the major histocompatibility complex) [43] and with enrichment analyses of MIR137 potential target pathways [44].

Of CHD8 gene sets, only the downregulated gene set showed evidence for significant association. CHD8 has not previously been centrally implicated in psychosis as it is associated with a congenital disorder (CHARGE syndrome) and linked to autism [26]. However, there is a reasonable argument to be made on the basis of common susceptibility to mental disorders that genes central to other major mental disorders might also affect schizophrenia. There has been recent evidence on rare variants in the gene [28, 45] being implicated in psychosis, which adds to the notion of the cross-disorder nature of CHD8 pathways. The downregulated gene set that was significantly associated with schizophrenia in the present study was also the one that Sugathan et al. [16] reported to be significantly enriched in autism.

In addition to the above findings, a systematic floor effect in polygenic scores was observed. We propose that this observation is consistent with predictions that would be made based on the recently proposed omnigenic model of complex traits such as schizophrenia. This model states that most genes expressed in cells that are relevant to the biology of an illness contribute to heritability and PRSs because of the likely interaction of multiple signalling pathways within cells that support their biological functions. In the light of this omnigenic hypothesis [12], implicating a greater number of SNPs than the ordinary polygenic model would suggest, our results support the hypothesis by demonstrating a weak polygenic effect extant in every random subset of genes. This omnigenic effect is also supported by Supplementary Fig. 2, which demonstrates an overall increase in SNP test statistics versus expected values, as well as the Q–Q plot in the original PGC2 report [1] that also showed a very similar effect across an increased number of observations. Enrichment analysis indicated an enrichment for broadly expressed genes, which also corroborates the principle finding of the omnigenic model [12] for schizophrenia. Additionally, we report that genic SNP sets seemed to explain slightly more variation than their non-genic counterparts. This indicates that studies implementing a pathway stratagem should be mindful of both effects when assessing if a gene set explains more variation than a random subset of genic or non-genic SNPs.

This study showed that several of the target putative core gene-sets investigated were highly significantly associated with schizophrenia, with the strongest effect being observed for the TCF4 core gene set. Even though most of the genes in these sets are not associated with risk in current GWAS datasets, they may be part of networks of genes that underlie common mechanisms for schizophrenia. These findings strongly indicate that, despite a very widespread, possibly even omnigenic contribution to risk, it is possible to identify subsets of genes making relatively larger contributions—putative core genes—which may implicate specific biochemical pathways or molecular processes with selectively greater roles in pathogenesis.

## Supplementary information

Supplementary Materials
