# Supplementary material for: The role of polygenic risk score gene-set analysis in the context of the omnigenic model of schizophrenia
Source: Neuropsychopharmacology. 2019 May 11;44(9):1562–9. doi: 10.1038/s41386-019-0410-z (PMC6785707; doi:10.1038/s41386-019-0410-z)
Supplement: Supplementary file 1 — Supplementary Materials [file 41386_2019_410_MOESM1_ESM.docx]

**Supplementary Information**

The members of the **Schizophrenia Working Group of the Psychiatric Genomics Consortium 2** are: Stephan Ripke, MD (Analytic and Translational Genetics Unit, Massachusetts General Hospital, Boston, Massachusetts 02114, USA and Stanley Center for Psychiatric Research, Broad Institute of MIT and Harvard, Cambridge, Massachusetts 02142, USA), Benjamin M Neale, PhD (Analytic and Translational Genetics Unit, Massachusetts General Hospital, Boston, Massachusetts 02114, USA and Psychiatric and Neurodevelopmental Genetics Unit, Massachusetts General Hospital, Boston, Massachusetts 02114, USA and Stanley Center for Psychiatric Research, Broad Institute of MIT and Harvard, Cambridge, Massachusetts 02142, USA and Medical and Population Genetics Program, Broad Institute of MIT and Harvard, Cambridge, Massachusetts 02142, USA), Aiden Corvin, MD,PhD (Neuropsychiatric Genetics Research Group, Department of Psychiatry, Trinity College Dublin, Dublin 8, Ireland), James TR Walters, MD,PhD (MRC Centre for Neuropsychiatric Genetics and Genomics, Institute of Psychological Medicine and Clinical Neurosciences, School of Medicine, Cardiff University, Cardiff, CF24 4HQ, UK), Kai-How Farh, MD,PhD (Analytic and Translational Genetics Unit, Massachusetts General Hospital, Boston, Massachusetts 02114, USA), Peter A Holmans, PhD (MRC Centre for Neuropsychiatric Genetics and Genomics, Institute of Psychological Medicine and Clinical Neurosciences, School of Medicine, Cardiff University, Cardiff, CF24 4HQ, UK and National Centre for Mental Health, Cardiff University, Cardiff, CF24 4HQ, UK), Phil Lee, PhD (Psychiatric and Neurodevelopmental Genetics Unit, Massachusetts General Hospital, Boston, Massachusetts 02114, USA and Analytic and Translational Genetics Unit, Massachusetts General Hospital, Boston, Massachusetts 02114, USA and Stanley Center for Psychiatric Research, Broad Institute of MIT and Harvard, Cambridge, Massachusetts 02142, USA), Brendan Bulik-Sullivan, BA (Analytic and Translational Genetics Unit, Massachusetts General Hospital, Boston, Massachusetts 02114, USA and Stanley Center for Psychiatric Research, Broad Institute of MIT and Harvard, Cambridge, Massachusetts 02142, USA), David A Collier, PhD (Eli Lilly and Company Limited, Erl Wood Manor, Sunninghill Road, Windlesham, Surrey, GU20 6PH, UK and Social, Genetic and Developmental Psychiatry Centre, Institute of Psychiatry, King's College London, London, SE5 8AF, UK), Hailiang Huang, PhD (Analytic and Translational Genetics Unit, Massachusetts General Hospital, Boston, Massachusetts 02114, USA and Medical and Population Genetics Program, Broad Institute of MIT and Harvard, Cambridge, Massachusetts 02142, USA), Tune H Pers, PhD (Center for Biological Sequence Analysis, Department of Systems Biology, Technical University of Denmark, DK-2800, Denmark and Medical and Population Genetics Program, Broad Institute of MIT and Harvard, Cambridge, Massachusetts 02142, USA and Division of Endocrinology and Center for Basic and Translational Obesity Research, Boston Children's Hospital, Boston, Massachusetts 02115, USA), Tune H Pers, PhD (Center for Biological Sequence Analysis, Department of Systems Biology, Technical University of Denmark, DK-2800, Denmark and Medical and Population Genetics Program, Broad Institute of MIT and Harvard, Cambridge, Massachusetts 02142, USA and Division of Endocrinology and Center for Basic and Translational Obesity Research, Boston Children's Hospital, Boston, Massachusetts 02115, USA), Ingrid Agartz, MD,PhD (NORMENT, KG Jebsen Centre for Psychosis Research, Institute of Clinical Medicine, University of Oslo, 0424 Oslo, Norway and Department of Psychiatry, Diakonhjemmet Hospital, 0319 Oslo, Norway and Department of Clinical Neuroscience, Psychiatry Section, Karolinska Institutet, SE-17176 Stockholm, Sweden ), Esben Agerbo, DMSc (National Centre for Register-based Research, Aarhus University, DK-8210 Aarhus, Denmark and Centre for Integrative Register-based Research, CIRRAU, Aarhus University, DK-8210 Aarhus, Denmark and The Lundbeck Foundation Initiative for Integrative Psychiatric Research, iPSYCH, Denmark), Margot Albus, MD (State Mental Hospital, 85540 Haar, Germany), Madeline Alexander, PhD (Department of Psychiatry and Behavioral Sciences, Stanford University, Stanford, California 94305, USA), Farooq Amin, MD (Department of Psychiatry and Behavioral Sciences, Emory University, Atlanta, Georgia 30322, USA and Department of Psychiatry and Behavioral Sciences, Atlanta Veterans Affairs Medical Center, Atlanta, Georgia 30033, USA), Silviu A Bacanu, PhD (Virginia Institute for Psychiatric and Behavioral Genetics, Department of Psychiatry, Virginia Commonwealth University, Richmond, Virginia 23298, USA), Martin Begemann, MD (Clinical Neuroscience, Max Planck Institute of Experimental Medicine, Göttingen 37075, Germany), Richard A Belliveau Jr, BA (Stanley Center for Psychiatric Research, Broad Institute of MIT and Harvard, Cambridge, Massachusetts 02142, USA), Judit Bene, PhD (Department of Medical Genetics, University of Pécs, Pécs H-7624, Hungary and Szentagothai Research Center, University of Pécs, Pécs H-7624, Hungary), Sarah E Bergen, PhD (Department of Medical Epidemiology and Biostatistics, Karolinska Institutet, Stockholm SE-17177, Sweden and Stanley Center for Psychiatric Research, Broad Institute of MIT and Harvard, Cambridge, Massachusetts 02142, USA), Elizabeth Bevilacqua, BS (Stanley Center for Psychiatric Research, Broad Institute of MIT and Harvard, Cambridge, Massachusetts 02142, USA), Tim B Bigdeli, PhD (Virginia Institute for Psychiatric and Behavioral Genetics, Department of Psychiatry, Virginia Commonwealth University, Richmond, Virginia 23298, USA), Donald W Black, MD (Department of Psychiatry, University of Iowa Carver College of Medicine, Iowa City, Iowa 52242, USA), Richard Bruggeman, MD,PhD (University Medical Center Groningen, Department of Psychiatry, University of Groningen, NL-9700 RB, The Netherlands), Nancy G Buccola, APRN,BC (School of Nursing, Louisiana State University Health Sciences Center, New Orleans, Louisiana 70112, USA), Randy L Buckner, PhD (Center for Brain Science, Harvard University, Cambridge, Massachusetts 02138, USA and Department of Psychiatry, Massachusetts General Hospital, Boston, Massachusetts 02114, USA and Athinoula A. Martinos Center, Massachusetts General Hospital, Boston, Massachusetts 02129, USA), William Byerley, MD (Department of Psychiatry, University of California at San Francisco, San Francisco, California, 94143 USA), Wiepke Cahn, MD,PhD (University Medical Center Utrecht, Department of Psychiatry, Rudolf Magnus Institute of Neuroscience, 3584 Utrecht, The Netherlands), Guiqing Cai, MD (Department of Psychiatry, Icahn School of Medicine at Mount Sinai, New York, New York 10029, USA and Department of Human Genetics, Icahn School of Medicine at Mount Sinai, New York, New York 10029, USA), Murray J Cairns, PhD (Schizophrenia Research Institute, Sydney NSW 2010, Australia and Priority Centre for Translational Neuroscience and Mental Health, University of Newcastle, Newcastle NSW 2300, Australia and School of Biomedical Sciences and Pharmacy, University of Newcastle, Callaghan NSW 2308, Australia), Dominique Campion, MD, PhD (Centre Hospitalier du Rouvray and INSERM U1079 Faculty of Medicine, 76301 Rouen, France), Rita M Cantor, PhD (Department of Human Genetics, David Geffen School of Medicine, University of California, Los Angeles, California 90095, USA), Vaughan J Carr, MD,FRANZCP,FRCPC (Schizophrenia Research Institute, Sydney NSW 2010, Australia and School of Psychiatry, University of New South Wales, Sydney NSW 2031, Australia), Noa Carrera, PhD (MRC Centre for Neuropsychiatric Genetics and Genomics, Institute of Psychological Medicine and Clinical Neurosciences, School of Medicine, Cardiff University, Cardiff, CF24 4HQ, UK), Stanley V Catts, MD,FRANZCP (Royal Brisbane and Women's Hospital, University of Queensland, Brisbane QLD 4072, Australia and Schizophrenia Research Institute, Sydney NSW 2010, Australia), Kimberley D Chambert, MSc (Stanley Center for Psychiatric Research, Broad Institute of MIT and Harvard, Cambridge, Massachusetts 02142, USA), Wei Cheng, (Department of Computer Science, University of North Carolina, Chapel Hill, North Carolina 27514, USA), C Robert Cloninger, MD, PhD (Department of Psychiatry, Washington University, St. Louis, Missouri 63110, USA), David Cohen, MD, PhD (Department of Child and Adolescent Psychiatry, Assistance Publique Hospitaux de Paris, Pierre and Marie Curie Faculty of Medicine and Institute for Intelligent Systems and Robotics, Paris, 75013, France), Nadine Cohen, PhD (Blue Note Biosciences, Princeton, New Jersey 08540, USA), Paul Cormican, PhD (Neuropsychiatric Genetics Research Group, Department of Psychiatry, Trinity College Dublin, Dublin 8, Ireland), Nick Craddock, MD,PhD (MRC Centre for Neuropsychiatric Genetics and Genomics, Institute of Psychological Medicine and Clinical Neurosciences, School of Medicine, Cardiff University, Cardiff, CF24 4HQ, UK and National Centre for Mental Health, Cardiff University, Cardiff, CF24 4HQ, UK), Benedicto Crespo-Facorro, MD, PhD (University Hospital Marqués de Valdecilla, Instituto de Formación e Investigación Marqués de Valdecilla, University of Cantabria, E‐39008 Santander, Spain and Centro Investigación Biomédica en Red Salud Mental, Madrid, Spain), James J Crowley, PhD (Department of Genetics, University of North Carolina, Chapel Hill, North Carolina 27599-7264, USA), David Curtis, MD,PhD,MRCPsych (Department of Psychological Medicine, Queen Mary University of London, London E1 1BB, UK and Molecular Psychiatry Laboratory, Division of Psychiatry, University College London, London WC1E 6JJ, UK), Michael Davidson, MD (Sheba Medical Center, Tel Hashomer 52621, Israel), Kenneth L Davis, MD (Department of Psychiatry, Icahn School of Medicine at Mount Sinai, New York, New York 10029, USA), Franziska Degenhardt, MD (Institute of Human Genetics, University of Bonn, D-53127 Bonn, Germany and Department of Genomics, Life and Brain Center, D-53127 Bonn, Germany), Jurgen Del Favero, PhD (Applied Molecular Genomics Unit, VIB Department of Molecular Genetics, University of Antwerp, B-2610 Antwerp, Belgium), Lynn E DeLisi, (VA Boston Health Care System, Brockton, Massachusetts 02301, USA and Department of Psychiatry, Harvard Medical School, Boston, Massachusetts 02115, USA), Ditte Demontis, MSc,PhD (Department of Biomedicine, Aarhus University, DK-8000 Aarhus C, Denmark and Centre for Integrative Sequencing, iSEQ, Aarhus University, DK-8000 Aarhus C, Denmark and The Lundbeck Foundation Initiative for Integrative Psychiatric Research, iPSYCH, Denmark), Dimitris Dikeos, MD (First Department of Psychiatry, University of Athens Medical School, Athens 11528, Greece), Timothy Dinan, MD,PhD (Department of Psychiatry, University College Cork, Co. Cork, Ireland), Srdjan Djurovic, PhD (NORMENT, KG Jebsen Centre for Psychosis Research, Institute of Clinical Medicine, University of Oslo, 0424 Oslo, Norway and Department of Medical Genetics, Oslo University Hospital, 0424 Oslo, Norway), Gary Donohoe , PhD (Cognitive Genetics and Therapy Group, School of Psychology and Discipline of Biochemistry, National University of Ireland Galway, Co. Galway, Ireland and Neuropsychiatric Genetics Research Group, Department of Psychiatry, Trinity College Dublin, Dublin 8, Ireland), Elodie Drapeau, PhD (Department of Psychiatry, Icahn School of Medicine at Mount Sinai, New York, New York 10029, USA), Jubao Duan, PhD (Department of Psychiatry and Behavioral Sciences, NorthShore University HealthSystem, Evanston, Illinois 60201, USA and Department of Psychiatry and Behavioral Neuroscience, University of Chicago, Chicago, Illinois 60637,, USA), Frank Dudbridge, PhD (Department of Non-Communicable Disease Epidemiology, London School of Hygiene and Tropical Medicine, London WC1E 7HT, UK), Peter Eichhammer, MD, PhD (Department of Psychiatry, University of Regensburg, 93053 Regensburg, Germany), Johan Eriksson, MD,PhD (Folkhälsan Research Center, Helsinki, Finland, Biomedicum Helsinki 1, Haartmaninkatu 8, FI-00290, Helsinki, Finland and National Institute for Health and Welfare, P.O. BOX 30, FI-00271 Helsinki, Finland and Department of General Practice, Helsinki University Central Hospital, University of Helsinki P.O. BOX 20, Tukholmankatu 8 B, FI-00014, Helsinki, Finland), Valentina Escott-Price, PhD (MRC Centre for Neuropsychiatric Genetics and Genomics, Institute of Psychological Medicine and Clinical Neurosciences, School of Medicine, Cardiff University, Cardiff, CF24 4HQ, UK), Laurent Essioux, PhD (Translational Technologies and Bioinformatics, Pharma Research and Early Development, F.Hoffman-La Roche, CH-4070 Basel, Switzerland), Ayman H Fanous, MD (Mental Health Service Line, Washington VA Medical Center, Washington DC 20422, USA and Department of Psychiatry, Georgetown University School of Medicine, Washington DC 20057, USA and Department of Psychiatry, Virginia Commonwealth University School of Medicine, Richmond, Virginia 23298, USA and Department of Psychiatry, Keck School of Medicine of the University of Southern California, Los Angeles, California 90033, USA), Martilias S Farrell, PhD (Department of Genetics, University of North Carolina, Chapel Hill, North Carolina 27599-7264, USA), Josef Frank, MSc (Department of Genetic Epidemiology in Psychiatry, Central Institute of Mental Health, Medical Faculty Mannheim, University of Heidelberg, Heidelberg, D-68159 Mannheim, Germany), Lude Franke, PhD (Department of Genetics, University of Groningen, University Medical Centre Groningen, 9700 RB Groningen, The Netherlands), Robert Freedman, MD (Department of Psychiatry, University of Colorado Denver, Aurora, Colorado 80045, USA), Nelson B Freimer, MD (Center for Neurobehavioral Genetics, Semel Institute for Neuroscience and Human Behavior, University of California, Los Angeles, California 90095, USA), Joseph I Friedman, MD (Department of Psychiatry, Icahn School of Medicine at Mount Sinai, New York, New York 10029, USA), Menachem Fromer, PhD (Division of Psychiatric Genomics, Department of Psychiatry, Icahn School of Medicine at Mount Sinai, New York, New York 10029, USA and Stanley Center for Psychiatric Research, Broad Institute of MIT and Harvard, Cambridge, Massachusetts 02142, USA and Psychiatric and Neurodevelopmental Genetics Unit, Massachusetts General Hospital, Boston, Massachusetts 02114, USA and Analytic and Translational Genetics Unit, Massachusetts General Hospital, Boston, Massachusetts 02114, USA), Giulio Genovese, PhD (Stanley Center for Psychiatric Research, Broad Institute of MIT and Harvard, Cambridge, Massachusetts 02142, USA), Lyudmila Georgieva , PhD (MRC Centre for Neuropsychiatric Genetics and Genomics, Institute of Psychological Medicine and Clinical Neurosciences, School of Medicine, Cardiff University, Cardiff, CF24 4HQ, UK), Elliot S Gershon, (Departments of Psychiatry and Human Genetics, University of Chicago, Chicago, Illinois 60637 USA), Ina Giegling, PhD (Department of Psychiatry, University of Halle, 06112 Halle, Germany and Department of Psychiatry, University of Munich, 80336, Munich, Germany), Paola Giusti-Rodríguez, PhD (Department of Genetics, University of North Carolina, Chapel Hill, North Carolina 27599-7264, USA), Stephanie Godard, MS (Departments of Psychiatry and Human and Molecular Genetics, INSERM, Institut de Myologie, Hôpital de la Pitiè-Salpêtrière, Paris, 75013, France), Jacqueline I Goldstein, SB (Analytic and Translational Genetics Unit, Massachusetts General Hospital, Boston, Massachusetts 02114, USA and Medical and Population Genetics Program, Broad Institute of MIT and Harvard, Cambridge, Massachusetts 02142, USA), Srihari Gopal, MD,MHS (Neuroscience Therapeutic Area, Janssen Research and Development, Raritan, New Jersey 08869, USA), Jacob Gratten, PhD (Queensland Brain Institute, The University of Queensland, Brisbane, QLD 4072, Australia), Lieuwe de Haan, MD,PhD (Academic Medical Centre University of Amsterdam, Department of Psychiatry, 1105 AZ Amsterdam, The Netherlands), Christian Hammer, PhD (Clinical Neuroscience, Max Planck Institute of Experimental Medicine, Göttingen 37075, Germany), Marian L Hamshere, PhD (MRC Centre for Neuropsychiatric Genetics and Genomics, Institute of Psychological Medicine and Clinical Neurosciences, School of Medicine, Cardiff University, Cardiff, CF24 4HQ, UK), Mark Hansen, PhD (Illumina, La Jolla, California, California 92122, USA), Thomas Hansen, MSc,PhD (Institute of Biological Psychiatry, Mental Health Centre Sct. Hans, Mental Health Services Copenhagen, DK-4000, Denmark and The Lundbeck Foundation Initiative for Integrative Psychiatric Research, iPSYCH, Denmark), Vahram Haroutunian, PhD (Department of Psychiatry, Icahn School of Medicine at Mount Sinai, New York, New York 10029, USA and J.J. Peters VA Medical Center, Bronx, New York, New York 10468, USA and Friedman Brain Institute, Icahn School of Medicine at Mount Sinai, New York, New York 10029, USA), Annette M Hartmann, PhD (Department of Psychiatry, University of Halle, 06112 Halle, Germany), Frans A Henskens, BMath,DipEd,DipCompSc,PhD (School of Electrical Engineering and Computer Science, University of Newcastle, Newcastle NSW 2308, Australia and Priority Research Centre for Health Behaviour, University of Newcastle, Newcastle NSW 2308, Australia and Schizophrenia Research Institute, Sydney NSW 2010, Australia), Joel N Hirschhorn, MD, PhD (Department of Genetics, Harvard Medical School, Boston, Massachusetts 02115, USA and Medical and Population Genetics Program, Broad Institute of MIT and Harvard, Cambridge, Massachusetts 02142, USA and Division of Endocrinology and Center for Basic and Translational Obesity Research, Boston Children's Hospital, Boston, Massachusetts 02115, USA), Mads V Hollegaard, MSc,PhD (Section of Neonatal Screening and Hormones, Department of Clinical Biochemistry, Immunology and Genetics, Statens Serum Institut, Copenhagen, DK-2300, Denmark), David M Hougaard, (Section of Neonatal Screening and Hormones, Department of Clinical Biochemistry, Immunology and Genetics, Statens Serum Institut, Copenhagen, DK-2300, Denmark), Inge Joa , PhD (Regional Centre for Clinical Research in Psychosis, Department of Psychiatry, Stavanger University Hospital, 4011 Stavanger, Norway), Antonio Julià, PhD (Rheumatology Research Group, Vall d'Hebron Research Institute, Barcelona, 08035, Spain), René S Kahn, MD,PhD (University Medical Center Utrecht, Department of Psychiatry, Rudolf Magnus Institute of Neuroscience, 3584 Utrecht, The Netherlands), Luba Kalaydjieva, MD,PhD (Centre for Medical Research, The University of Western Australia, Perth, WA 6009, Australia and The Perkins Institute for Medical Research, The University of Western Australia, Perth, WA 6009, Australia ), Sena Karachanak-Yankova, PhD (Department of Medical Genetics, Medical University, Sofia 1431, Bulgaria), Juha Karjalainen, PhD (Department of Genetics, University of Groningen, University Medical Centre Groningen, 9700 RB Groningen, The Netherlands), David Kavanagh, PhD (MRC Centre for Neuropsychiatric Genetics and Genomics, Institute of Psychological Medicine and Clinical Neurosciences, School of Medicine, Cardiff University, Cardiff, CF24 4HQ, UK), Matthew C Keller, PhD (Department of Psychology, University of Colorado Boulder, Boulder, Colorado 80309, USA), Brian J Kelly, PhD, FRANZCP (Priority Centre for Translational Neuroscience and Mental Health, University of Newcastle, Newcastle NSW 2300, Australia), James L Kennedy, MSc,MD,FRCPC (Campbell Family Mental Health Research Institute, Centre for Addiction and Mental Health, Toronto, Ontario, M5T 1R8, Canada and Department of Psychiatry, University of Toronto, Toronto, Ontario, M5T 1R8, Canada and Institute of Medical Science, University of Toronto, Toronto, Ontario, M5S 1A8, Canada), Andrey Khrunin, PhD (Institute of Molecular Genetics, Russian Academy of Sciences, Moscow 123182, Russia), Yunjung Kim, PhD (Department of Genetics, University of North Carolina, Chapel Hill, North Carolina 27599-7264, USA), Janis Klovins, PhD (Latvian Biomedical Research and Study Centre, Riga, LV-1067, Latvia), James A Knowles, MD,PhD (Department of Psychiatry and Zilkha Neurogenetics Institute, Keck School of Medicine at University of Southern California, Los Angeles, California 90089, USA), Bettina Konte, MSc (Department of Psychiatry, University of Halle, 06112 Halle, Germany), Vaidutis Kucinskas, PhD (Faculty of Medicine, Vilnius University, LT-01513 Vilnius, Lithuania), Zita Ausrele Kucinskiene, MD,PhD (Faculty of Medicine, Vilnius University, LT-01513 Vilnius, Lithuania), Hana Kuzelova-Ptackova, PhD (Department of Biology and Medical Genetics, 2nd Faculty of Medicine and University Hospital Motol, 150 06 Prague, Czech Republic), Anna K Kähler, PhD (Department of Medical Epidemiology and Biostatistics, Karolinska Institutet, Stockholm SE-17177, Sweden), Claudine Laurent, MD, PhD (Department of Psychiatry and Behavioral Sciences, Stanford University, Stanford, California 94305, USA and Department of Child and Adolescent Psychiatry, Pierre and Marie Curie Faculty of Medicine, Paris 75013, France), S Hong Lee, PhD (Queensland Brain Institute, The University of Queensland, Brisbane, QLD 4072, Australia), Sophie E Legge, BSc (MRC Centre for Neuropsychiatric Genetics and Genomics, Institute of Psychological Medicine and Clinical Neurosciences, School of Medicine, Cardiff University, Cardiff, CF24 4HQ, UK), Bernard Lerer, MD (Department of Psychiatry, Hadassah-Hebrew University Medical Center, Jerusalem 91120, Israel), Kung-Yee Liang, PhD (Department of Biostatistics, Johns Hopkins University Bloomberg School of Public Health, Baltimore, Maryland 21205, USA), Jeffrey Lieberman, (Department of Psychiatry, Columbia University, New York, New York 10032, USA), Svetlana Limborska, PhD (Institute of Molecular Genetics, Russian Academy of Sciences, Moscow 123182, Russia), Carmel M Loughland, BA(Hons), PhD (Schizophrenia Research Institute, Sydney NSW 2010, Australia and Priority Centre for Translational Neuroscience and Mental Health, University of Newcastle, Newcastle NSW 2300, Australia), Jan Lubinski, MD,PhD (Department of Genetics and Pathology, International Hereditary Cancer Center, Pomeranian Medical University in Szczecin, 70-453 Szczecin, Poland), Jouko Lönnqvist, MD,PhD (Department of Mental Health and Substance Abuse Services; National Institute for Health and Welfare, P.O. BOX 30, FI-00271 Helsinki, Finland), Milan Macek Jr, MD,PhD (Department of Biology and Medical Genetics, 2nd Faculty of Medicine and University Hospital Motol, 150 06, Prague, Czech Republic), Patrik KE Magnusson, PhD (Department of Medical Epidemiology and Biostatistics, Karolinska Institutet, Stockholm SE-17177, Sweden), Brion S Maher, PhD (Department of Mental Health, Bloomberg School of Public Health, Johns Hopkins University, Baltimore, Maryland 21205, USA), Wolfgang Maier, MD (Department of Psychiatry, University of Bonn, D-53127 Bonn, Germany), Jacques Mallet, PhD (Centre National de la Recherche Scientifique, Laboratoire de Génétique Moléculaire de la Neurotransmission et des Processus Neurodégénératifs, Hôpital de la Pitié Salpêtrière, 75013, Paris, France), Sara Marsal, MD,PhD (Rheumatology Research Group, Vall d'Hebron Research Institute, Barcelona, 08035, Spain), Manuel Mattheisen, MD (Department of Biomedicine, Aarhus University, DK-8000 Aarhus C, Denmark and Centre for Integrative Sequencing, iSEQ, Aarhus University, DK-8000 Aarhus C, Denmark and The Lundbeck Foundation Initiative for Integrative Psychiatric Research, iPSYCH, Denmark and Department of Genomics Mathematics, University of Bonn, D-53127 Bonn, Germany), Morten Mattingsdal, MS (NORMENT, KG Jebsen Centre for Psychosis Research, Institute of Clinical Medicine, University of Oslo, 0424 Oslo, Norway and Research Unit, Sørlandet Hospital, 4604 Kristiansand, Norway), Robert W McCarley, MD (VA Boston Health Care System, Brockton, Massachusetts 02301, USA and Department of Psychiatry, Harvard Medical School, Boston, Massachusetts 02115, USA), Colm McDonald, MD,PhD (Department of Psychiatry, National University of Ireland Galway, Co. Galway, Ireland ), Andrew M McIntosh, MD,FRCPsych (Division of Psychiatry, University of Edinburgh, Edinburgh EH16 4SB, UK and Centre for Cognitive Ageing and Cognitive Epidemiology, University of Edinburgh, Edinburgh EH16 4SB, UK), Carin J Meijer, PhD (Academic Medical Centre University of Amsterdam, Department of Psychiatry, 1105 AZ Amsterdam, The Netherlands), Bela Melegh, MD,PhD,DSc (Department of Medical Genetics, University of Pécs, Pécs H-7624, Hungary and Szentagothai Research Center, University of Pécs, Pécs H-7624, Hungary), Ingrid Melle, MD,PhD (NORMENT, KG Jebsen Centre for Psychosis Research, Institute of Clinical Medicine, University of Oslo, 0424 Oslo, Norway and Division of Mental Health and Addiction, Oslo University Hospital, 0424 Oslo, Norway), Raquelle I Mesholam-Gately, PhD (Massachusetts Mental Health Center Public Psychiatry Division of the Beth Israel Deaconess Medical Center, Boston, Massachusetts 02114, USA and Department of Psychiatry, Harvard Medical School, Boston, Massachusetts 02115, USA), Andres Metspalu, MD,PhD (Estonian Genome Center, University of Tartu, Tartu 50090, Estonia), Patricia T Michie, PhD (School of Psychology, University of Newcastle, Newcastle NSW 2308, Australia and Schizophrenia Research Institute, Sydney NSW 2010, Australia), Lili Milani, PhD (Estonian Genome Center, University of Tartu, Tartu 50090, Estonia), Vihra Milanova, MD,PhD (First Psychiatric Clinic, Medical University, Sofia 1431, Bulgaria), Younes Mokrab, PhD (Eli Lilly and Company Limited, Erl Wood Manor, Sunninghill Road, Windlesham, Surrey, GU20 6PH UK), Derek W Morris, PhD (Cognitive Genetics and Therapy Group, School of Psychology and Discipline of Biochemistry, National University of Ireland Galway, Co. Galway, Ireland and Neuropsychiatric Genetics Research Group, Department of Psychiatry, Trinity College Dublin, Dublin 8, Ireland), Ole Mors, MD,PhD (Department P, Aarhus University Hospital, DK-8240 Risskov, Denmark and Centre for Integrative Sequencing, iSEQ, Aarhus University, DK-8000 Aarhus C, Denmark and The Lundbeck Foundation Initiative for Integrative Psychiatric Research, iPSYCH, Denmark), Kieran C Murphy, MD,PhD (Department of Psychiatry, Royal College of Surgeons in Ireland, Dublin 2, Ireland), Robin M Murray, FRS (King's College London, London SE5 8AF, UK), Inez Myin-Germeys, PhD (Maastricht University Medical Centre, South Limburg Mental Health Research and Teaching Network, EURON, 6229 HX Maastricht, The Netherlands), Mari Nelis, PhD (Estonian Genome Center, University of Tartu, Tartu 50090, Estonia), Deborah A Nertney, BSc (Queensland Centre for Mental Health Research, University of Queensland, Brisbane QLD 4076, Australia), Gerald Nestadt, MBBCh MPH (Department of Psychiatry and Behavioral Sciences, Johns Hopkins University School of Medicine, Baltimore, Maryland 21205, USA), Kristin K Nicodemus, PhD (Department of Psychiatry, Trinity College Dublin, Dublin 2, Ireland), Liene Nikitina-Zake, MD,PhD (Latvian Biomedical Research and Study Centre, Riga, LV-1067, Latvia), Laura Nisenbaum, PhD (Eli Lilly and Company, Lilly Corporate Center, Indianapolis, 46285 Indiana, USA), Annelie Nordin, (Department of Clinical Sciences, Psychiatry, Umeå University, SE-901 87 Umeå, Sweden), Colm O'Dushlaine, PhD (Stanley Center for Psychiatric Research, Broad Institute of MIT and Harvard, Cambridge, Massachusetts 02142, USA), F Anthony O'Neill, MD,PhD (Centre for Public Health, Institute of Clinical Sciences, Queen's University Belfast, Belfast BT12 6AB, UK), Sang-Yun Oh, PhD (Lawrence Berkeley National Laboratory, University of California at Berkeley, Berkeley, California 94720, USA), Ann Olincy, MD (Department of Psychiatry, University of Colorado Denver, Aurora, Colorado 80045, USA), Line Olsen, MSc,PhD (Institute of Biological Psychiatry, Mental Health Centre Sct. Hans, Mental Health Services Copenhagen, DK-4000, Denmark and The Lundbeck Foundation Initiative for Integrative Psychiatric Research, iPSYCH, Denmark), Jim Van Os, MD,PhD (Institute of Psychiatry, King's College London, London SE5 8AF, UK and Maastricht University Medical Centre, South Limburg Mental Health Research and Teaching Network, EURON, 6229 HX Maastricht, The Netherlands), Christos Pantelis, MD,MRCPsych,FRANZCP (Melbourne Neuropsychiatry Centre, University of Melbourne & Melbourne Health, Melbourne VIC 3053, Australia and Schizophrenia Research Institute, Sydney NSW 2010, Australia), George N Papadimitriou, MD (First Department of Psychiatry, University of Athens Medical School, Athens 11528, Greece), Sergi Papiol, PhD (Clinical Neuroscience, Max Planck Institute of Experimental Medicine, Göttingen 37075, Germany), Elena Parkhomenko, PhD (Department of Psychiatry, Icahn School of Medicine at Mount Sinai, New York, New York 10029, USA), Michele T Pato, MD (Department of Psychiatry and Zilkha Neurogenetics Institute, Keck School of Medicine at University of Southern California, Los Angeles, California 90089, USA), Tiina Paunio, MD,PhD (Public Health Genomics Unit, National Institute for Health and Welfare, P.O. BOX 30, FI-00271 Helsinki, Finland and Department of Psychiatry, University of Helsinki, P.O BOX 590, FI-00029 HUS, Helsinki, Finland), Diana O Perkins, MD, MPH (Department of Psychiatry, University of North Carolina, Chapel Hill, North Carolina 27599-7160, USA), Olli Pietiläinen, MSc (Institute for Molecular Medicine Finland, FIMM, University of Helsinki, P.O. BOX 20 FI-00014, Helsinki, Finland and Public Health Genomics Unit, National Institute for Health and Welfare, P.O. BOX 30, FI-00271 Helsinki, Finland), Jonathan Pimm, MB,BS,MD,MRCPsych (Molecular Psychiatry Laboratory, Division of Psychiatry, University College London, London WC1E 6JJ, UK), Andrew J Pocklington, PhD (MRC Centre for Neuropsychiatric Genetics and Genomics, Institute of Psychological Medicine and Clinical Neurosciences, School of Medicine, Cardiff University, Cardiff, CF24 4HQ, UK), John Powell, PhD (King's College London, London SE5 8AF, UK), Alkes Price, PhD (Department of Epidemiology, Harvard School of Public Health, Boston, Massachusetts 02115, USA and Medical and Population Genetics Program, Broad Institute of MIT and Harvard, Cambridge, Massachusetts 02142, USA), Ann E Pulver, PhD (Department of Psychiatry and Behavioral Sciences, Johns Hopkins University School of Medicine, Baltimore, Maryland 21205, USA), Shaun M Purcell, PhD (Division of Psychiatric Genomics, Department of Psychiatry, Icahn School of Medicine at Mount Sinai, New York, New York 10029, USA), Digby Quested, MBChB,MD,FRCPsych (Department of Psychiatry, University of Oxford, Oxford, OX3 7JX, UK), Henrik B Rasmussen, DVM,PhD (Institute of Biological Psychiatry, Mental Health Centre Sct. Hans, Mental Health Services Copenhagen, DK-4000, Denmark and The Lundbeck Foundation Initiative for Integrative Psychiatric Research, iPSYCH, Denmark), Abraham Reichenberg, PhD (Department of Psychiatry, Icahn School of Medicine at Mount Sinai, New York, New York 10029, USA and Friedman Brain Institute, Icahn School of Medicine at Mount Sinai, New York, NY, USA), Mark A Reimers, PhD (Virginia Institute for Psychiatric and Behavioral Genetics, Virginia Commonwealth University, Richmond, Virginia 23298, USA), Alexander L Richards, PhD (MRC Centre for Neuropsychiatric Genetics and Genomics, Institute of Psychological Medicine and Clinical Neurosciences, School of Medicine, Cardiff University, Cardiff, CF24 4HQ, UK and National Centre for Mental Health, Cardiff University, Cardiff, CF24 4HQ, UK), Joshua L Roffman, MD, MMSc (Department of Psychiatry, Massachusetts General Hospital, Boston, Massachusetts 02114, USA and Athinoula A. Martinos Center, Massachusetts General Hospital, Boston, Massachusetts 02129, USA), Panos Roussos, MD,PhD (Division of Psychiatric Genomics, Department of Psychiatry, Icahn School of Medicine at Mount Sinai, New York, New York 10029, USA and Institute for Multiscale Biology, Icahn School of Medicine at Mount Sinai, New York, New York 10029, USA), Douglas M Ruderfer, PhD (Division of Psychiatric Genomics, Department of Psychiatry, Icahn School of Medicine at Mount Sinai, New York, New York 10029, USA and MRC Centre for Neuropsychiatric Genetics and Genomics, Institute of Psychological Medicine and Clinical Neurosciences, School of Medicine, Cardiff University, Cardiff, CF24 4HQ, UK), Veikko Salomaa, MD,PhD (National Institute for Health and Welfare, P.O BOX 30, FI-00271 Helsinki, Finland), Alan R Sanders, MD (Department of Psychiatry and Behavioral Sciences, NorthShore University HealthSystem, Evanston, Illinois 60201, USA and Department of Psychiatry and Behavioral Neuroscience, University of Chicago, Chicago, Illinois 60637, USA), Ulrich Schall, MD,FRANZCP,PhD,DSc (Priority Centre for Translational Neuroscience and Mental Health, University of Newcastle, Newcastle NSW 2300, Australia and Schizophrenia Research Institute, Sydney NSW 2010, Australia), Sibylle G Schwab, PhD (Faculty of Science, Medicine & Health, Univeristy of Wollogong, NSW 2522 Australia), Edward M Scolnick, MD (Stanley Center for Psychiatric Research, Broad Institute of MIT and Harvard, Cambridge, Massachusetts 02142, USA), Rodney J Scott, PhD,FRCPath,FHGSA,FFSc (School of Biomedical Sciences and Pharmacy, University of Newcastle, Callaghan NSW 2308, Australia and Hunter New England Health Service, Newcastle NSW 2308, Australia and Schizophrenia Research Institute, Sydney NSW 2010, Australia), Larry J Seidman, PhD (Massachusetts Mental Health Center Public Psychiatry Division of the Beth Israel Deaconess Medical Center, Boston, Massachusetts 02114, USA and Department of Psychiatry, Harvard Medical School, Boston, Massachusetts 02115, USA), Jianxin Shi, PhD (Division of Cancer Epidemiology and Genetics, National Cancer Institute, Bethesda, Maryland 20892, USA), Jeremy M Silverman, PhD (Department of Psychiatry, Icahn School of Medicine at Mount Sinai, New York, New York 10029, USA and Research and Development, Bronx Veterans Affairs Medical Center, New York, New York 10468, USA), Petr Slominsky, PhD (Institute of Molecular Genetics, Russian Academy of Sciences, Moscow 123182, Russia), Jordan W Smoller, MD, ScD (Psychiatric and Neurodevelopmental Genetics Unit, Massachusetts General Hospital, Boston, Massachusetts 02114, USA and Stanley Center for Psychiatric Research, Broad Institute of MIT and Harvard, Cambridge, Massachusetts 02142, USA), Chris C A Spencer, PhD (Wellcome Trust Centre for Human Genetics, Oxford, OX3 7BN, UK), Eli A Stahl, PhD (Division of Psychiatric Genomics, Department of Psychiatry, Icahn School of Medicine at Mount Sinai, New York, New York 10029, USA and Medical and Population Genetics Program, Broad Institute of MIT and Harvard, Cambridge, Massachusetts 02142, USA), Elisabeth Stogmann , MD (Department of Clinical Neurology, Medical University of Vienna, 1090 Wien, Austria), Richard E Straub, PhD (Lieber Institute for Brain Development, Baltimore, Maryland 21205, USA), Eric Strengman, BSc (Department of Medical Genetics, University Medical Centre Utrecht, Universiteitsweg 100, 3584 CG, Utrecht, The Netherlands and University Medical Center Utrecht, Department of Psychiatry, Rudolf Magnus Institute of Neuroscience, 3584 Utrecht, The Netherlands), T Scott Stroup, MD, MPH (Department of Psychiatry, Columbia University, New York, New York 10032, USA), Jaana Suvisaari, MD,PhD (Department of Mental Health and Substance Abuse Services; National Institute for Health and Welfare, P.O. BOX 30, FI-00271 Helsinki, Finland), Dragan M Svrakic, MD,PhD (Department of Psychiatry, Washington University, St. Louis, Missouri 63110, USA), Jin P Szatkiewicz, PhD (Department of Genetics, University of North Carolina, Chapel Hill, North Carolina 27599-7264, USA), Erik Söderman, PhD (Department of Clinical Neuroscience, Psychiatry Section, Karolinska Institutet, SE-17176 Stockholm, Sweden), Srinivas Thirumalai, MB,BS,MD,MRCPsycH (Berkshire Healthcare NHS Foundation Trust, Bracknell RG12 1BQ, UK), Draga Toncheva, MD,PhD,DSc (Department of Medical Genetics, Medical University, Sofia1431, Bulgaria), Paul A Tooney, PhD (School of Biomedical Sciences and Pharmacy, University of Newcastle, Callaghan NSW 2308, Australia and Priority Research Centre for Translational Neuroscience and Mental Health, University of Newcastle, Newcastle NSW 2300, Australia and Schizophrenia Research Institute, Sydney NSW 2010, Australia), John Waddington, PhD,DSc (Molecular and Cellular Therapeutics, Royal College of Surgeons in Ireland, Dublin 2, Ireland), Dermot Walsh, MD (Health Research Board, Dublin 2, Ireland), Dai Wang, PhD (Neuroscience Therapeutic Area, Janssen Research and Development, Raritan, New Jersey 08869, USA), Bradley T Webb, PhD (Virginia Institute for Psychiatric and Behavioral Genetics, Department of Psychiatry, Virginia Commonwealth University, Richmond, Virginia 23298, USA), Mark Weiser, MD (Sheba Medical Center, Tel Hashomer 52621, Israel), Dieter B Wildenauer, PhD (School of Psychiatry and Clinical Neurosciences, The University of Western Australia, Perth WA 6009, Australia), Nigel M Williams, PhD (MRC Centre for Neuropsychiatric Genetics and Genomics, Institute of Psychological Medicine and Clinical Neurosciences, School of Medicine, Cardiff University, Cardiff, CF24 4HQ, UK), Stephanie Williams, ScM (Department of Genetics, University of North Carolina, Chapel Hill, North Carolina 27599-7264, USA), Aaron R Wolen, PhD (Virginia Institute for Psychiatric and Behavioral Genetics, Virginia Commonwealth University, Richmond, Virginia 23298, USA), Brandon K Wormley, BA (Virginia Institute for Psychiatric and Behavioral Genetics, Department of Psychiatry, Virginia Commonwealth University, Richmond, Virginia 23298, USA), Jing Qin Wu, PhD (School of Biomedical Sciences and Pharmacy, University of Newcastle, Callaghan NSW 2308, Australia and Schizophrenia Research Institute, Sydney NSW 2010, Australia.), Clement C Zai, PhD (Campbell Family Mental Health Research Institute, Centre for Addiction and Mental Health, Toronto, Ontario, M5T 1R8, Canada and Department of Psychiatry, University of Toronto, Toronto, Ontario, M5T 1R8, Canada), Fritz Zimprich, MD,PhD (Department of Clinical Neurology, Medical University of Vienna, 1090 Wien, Austria), Rolf Adolfsson, MD,PhD (Department of Clinical Sciences, Psychiatry, Umeå University, SE-901 87 Umeå, Sweden), Ole A Andreassen, MD,PhD (NORMENT, KG Jebsen Centre for Psychosis Research, Institute of Clinical Medicine, University of Oslo, 0424 Oslo, Norway and Division of Mental Health and Addiction, Oslo University Hospital, 0424 Oslo, Norway), Douglas HR Blackwood, PhD,FRCPsych (Division of Psychiatry, University of Edinburgh, Edinburgh EH16 4SB, UK), Elvira Bramon, MD,PhD (University College London, London WC1E 6BT, UK), Joseph D Buxbaum, PhD (Department of Psychiatry, Icahn School of Medicine at Mount Sinai, New York, New York 10029, USA and Department of Human Genetics, Icahn School of Medicine at Mount Sinai, New York, New York 10029, USA and Department of Neuroscience, Icahn School of Medicine at Mount Sinai, New York, New York 10029, USA and Friedman Brain Institute, Icahn School of Medicine at Mount Sinai, New York, New York 10029, USA), Anders D Børglum, MD,PhD (Department of Biomedicine, Aarhus University, DK-8000 Aarhus C, Denmark and Centre for Integrative Sequencing, iSEQ, Aarhus University, DK-8000 Aarhus C, Denmark and Department P, Aarhus University Hospital, DK-8240 Risskov, Denmark and The Lundbeck Foundation Initiative for Integrative Psychiatric Research, iPSYCH, Denmark), Tõnu Esko, PhD (Estonian Genome Center, University of Tartu, Tartu 50090, Estonia and Division of Endocrinology and Center for Basic and Translational Obesity Research, Boston Children's Hospital, Boston, Massachusetts 02115, USA and Department of Genetics, Harvard Medical School, Boston, Massachusetts 02115, USA and Medical and Population Genetics Program, Broad Institute of MIT and Harvard, Cambridge, Massachusetts 02142, USA), Pablo V Gejman, MD (Department of Psychiatry and Behavioral Sciences, NorthShore University HealthSystem, Evanston, Illinois 60201, USA and Department of Psychiatry and Behavioral Neuroscience, University of Chicago, Chicago, Illinois 60637, USA), Michael Gill , MRCPsych,MD (Neuropsychiatric Genetics Research Group, Department of Psychiatry, Trinity College Dublin, Dublin 8, Ireland), Christina M Hultman, PhD (Department of Medical Epidemiology and Biostatistics, Karolinska Institutet, Stockholm SE-17177, Sweden), Assen V Jablensky, MD,DMSc,FRANZCP,FRCPsych (School of Psychiatry and Clinical Neurosciences, The University of Western Australia, Perth, WA 6009, Australia and Centre for Clinical Research in Neuropsychiatry, School of Psychiatry and Clinical Neurosciences, The University of Western Australia, Medical Research Foundation Building, Perth WA 6000, Australia and The Perkins Institute for Medical Research,The University of Western Australia, Perth, WA 6009, Australia and Schizophrenia Research Institute, Sydney NSW 2010, Australia), Erik G Jönsson, MD,PhD (Department of Clinical Neuroscience, Psychiatry Section, Karolinska Institutet, SE-17176 Stockholm, Sweden and NORMENT, KG Jebsen Centre for Psychosis Research, Institute of Clinical Medicine, University of Oslo, 0424 Oslo, Norway), Kenneth S Kendler, MD (Virginia Institute for Psychiatric and Behavioral Genetics, Departments of Psychiatry and Human and Molecular Genetics, Virginia Commonwealth University, Richmond, Virginia 23298, USA), George Kirov, MD,PhD (MRC Centre for Neuropsychiatric Genetics and Genomics, Institute of Psychological Medicine and Clinical Neurosciences, School of Medicine, Cardiff University, Cardiff, CF24 4HQ, UK), Jo Knight, PhD (Campbell Family Mental Health Research Institute, Centre for Addiction and Mental Health, Toronto, Ontario, M5T 1R8, Canada and Department of Psychiatry, University of Toronto, Toronto, Ontario, M5T 1R8, Canada and Institute of Medical Science, University of Toronto, Toronto, Ontario, M5S 1A8, Canada), Todd Lencz, PhD (Hofstra Northwell School of Medicine, Hempstead, New York 11549, USA and The Feinstein Institute for Medical Research, Manhasset, New York 11030, USA and The Hofstra NS-LIJ School of Medicine, Hempstead, New York 11549, USA), Douglas F Levinson, MD (Department of Psychiatry and Behavioral Sciences, Stanford University, Stanford, California 94305, USA), Qingqin S Li, PhD (Neuroscience Therapeutic Area, Janssen Research and Development, Raritan, New Jersey 08869, USA), Jianjun Liu, PhD (Human Genetics, Genome Institute of Singapore, A*STAR, Singapore 138672, Singapore and Saw Swee Hock School of Public Health, National University of Singapore, Singapore 117597, Singapore), Anil K Malhotra, MD (The Zucker Hillside Hospital, Glen Oaks, New York 11004, USA and The Feinstein Institute for Medical Research, Manhasset, New York 11030, USA and The Hofstra NS-LIJ School of Medicine, Hempstead, New York 11549, USA), Steven A McCarroll, PhD (Stanley Center for Psychiatric Research, Broad Institute of MIT and Harvard, Cambridge, Massachusetts 02142, USA and Department of Genetics, Harvard Medical School, Boston, Massachusetts 02115, USA), Andrew McQuillin, PhD (Molecular Psychiatry Laboratory, Division of Psychiatry, University College London, London WC1E 6JJ, UK), Jennifer L Moran, PhD (Stanley Center for Psychiatric Research, Broad Institute of MIT and Harvard, Cambridge, Massachusetts 02142, USA), Preben B Mortensen, MD,DMSc (National Centre for Register-based Research, Aarhus University, DK-8210 Aarhus, Denmark and Centre for Integrative Register-based Research, CIRRAU, Aarhus University, DK-8210 Aarhus, Denmark and The Lundbeck Foundation Initiative for Integrative Psychiatric Research, iPSYCH, Denmark), Bryan J Mowry, MB, BS, MD, FRANZCP (Queensland Brain Institute, The University of Queensland, Brisbane QLD 4072, Australia and Queensland Centre for Mental Health Research, University of Queensland, Brisbane QLD 4076, Australia), Roel A Ophoff, PhD (Center for Neurobehavioral Genetics, Semel Institute for Neuroscience and Human Behavior, University of California, Los Angeles, California 90095, USA and Department of Human Genetics, David Geffen School of Medicine, University of California, Los Angeles, California 90095, USA and University Medical Center Utrecht, Department of Psychiatry, Rudolf Magnus Institute of Neuroscience, 3584 Utrecht, The Netherlands), Michael J Owen, MD,PhD (MRC Centre for Neuropsychiatric Genetics and Genomics, Institute of Psychological Medicine and Clinical Neurosciences, School of Medicine, Cardiff University, Cardiff, CF24 4HQ, UK and National Centre for Mental Health, Cardiff University, Cardiff, CF24 4HQ, UK), Aarno Palotie, MD,PhD (Institute for Molecular Medicine Finland, FIMM, University of Helsinki, P.O. BOX 20, FI-00014, Helsinki, Finland and Stanley Center for Psychiatric Research, Broad Institute of MIT and Harvard, Cambridge, Massachusetts 02142, USA and Psychiatric and Neurodevelopmental Genetics Unit, Massachusetts General Hospital, Boston, Massachusetts 02114, USA), Carlos N Pato, MD,PhD (Department of Psychiatry and Zilkha Neurogenetics Institute, Keck School of Medicine at University of Southern California, Los Angeles, California 90089, USA), Tracey L Petryshen, PhD (Center for Human Genetic Research and Department of Psychiatry, Massachusetts General Hospital, Boston, Massachusetts 02114, USA and Department of Psychiatry, Harvard Medical School, Boston, Massachusetts 02115, USA and Stanley Center for Psychiatric Research, Broad Institute of MIT and Harvard, Cambridge, Massachusetts 02142, USA), Danielle Posthuma, PhD (Department of Functional Genomics, Center for Neurogenomics and Cognitive Research, Neuroscience Campus Amsterdam, VU University, Amsterdam 1081, The Netherlands and Department of Complex Trait Genetics, Neuroscience Campus Amsterdam, VU University Medical Center Amsterdam, Amsterdam 1081, The Netherlands and Department of Child and Adolescent Psychiatry, Erasmus University Medical Centre, Rotterdam 3000, The Netherlands), Brien P Riley, PhD (Virginia Institute for Psychiatric and Behavioral Genetics, Departments of Psychiatry and Human and Molecular Genetics, Virginia Commonwealth University, Richmond, Virginia 23298, USA), Pamela Sklar, MD,PhD (Division of Psychiatric Genomics, Department of Psychiatry, Icahn School of Medicine at Mount Sinai, New York, New York 10029, USA and Institute for Multiscale Biology, Icahn School of Medicine at Mount Sinai, New York, New York 10029, USA and Friedman Brain Institute, Icahn School of Medicine at Mount Sinai, New York, New York 10029, USA), David St Clair, MD, PhD (University of Aberdeen, Institute of Medical Sciences, Aberdeen, AB25 2ZD, UK), Daniel R Weinberger, MD (Lieber Institute for Brain Development, Baltimore, Maryland 21205, USA and Departments of Psychiatry, Neurology, Neuroscience and Institute of Genetic Medicine, Johns Hopkins School of Medicine, Baltimore, Maryland 21205, USA ), Thomas Werge, MSc,PhD (Institute of Biological Psychiatry, Mental Health Centre Sct. Hans, Mental Health Services Copenhagen, DK-4000, Denmark and Department of Clinical Medicine, University of Copenhagen, Copenhagen 2200, Denmark and The Lundbeck Foundation Initiative for Integrative Psychiatric Research, iPSYCH, Denmark), Mark J Daly, PhD (Analytic and Translational Genetics Unit, Massachusetts General Hospital, Boston, Massachusetts 02114, USA and Stanley Center for Psychiatric Research, Broad Institute of MIT and Harvard, Cambridge, Massachusetts 02142, USA and Medical and Population Genetics Program, Broad Institute of MIT and Harvard, Cambridge, Massachusetts 02142, USA), Patrick F Sullivan, MD,FRANZCP (Department of Genetics, University of North Carolina, Chapel Hill, North Carolina 27599-7264, USA and Department of Psychiatry, University of North Carolina, Chapel Hill, North Carolina 27599-7160, USA and Department of Medical Epidemiology and Biostatistics, Karolinska Institutet, Stockholm SE-17177, Sweden), Michael C O'Donovan, MD,PhD (MRC Centre for Neuropsychiatric Genetics and Genomics, Institute of Psychological Medicine and Clinical Neurosciences, School of Medicine, Cardiff University, Cardiff, CF24 4HQ, UK and National Centre for Mental Health, Cardiff University, Cardiff, CF24 4HQ, UK).

All authors declare no biomedical financial interests or potential conflicts of interest.

**Supplementary Figure S1. Graphic representation of the Leave One Out permutation process**


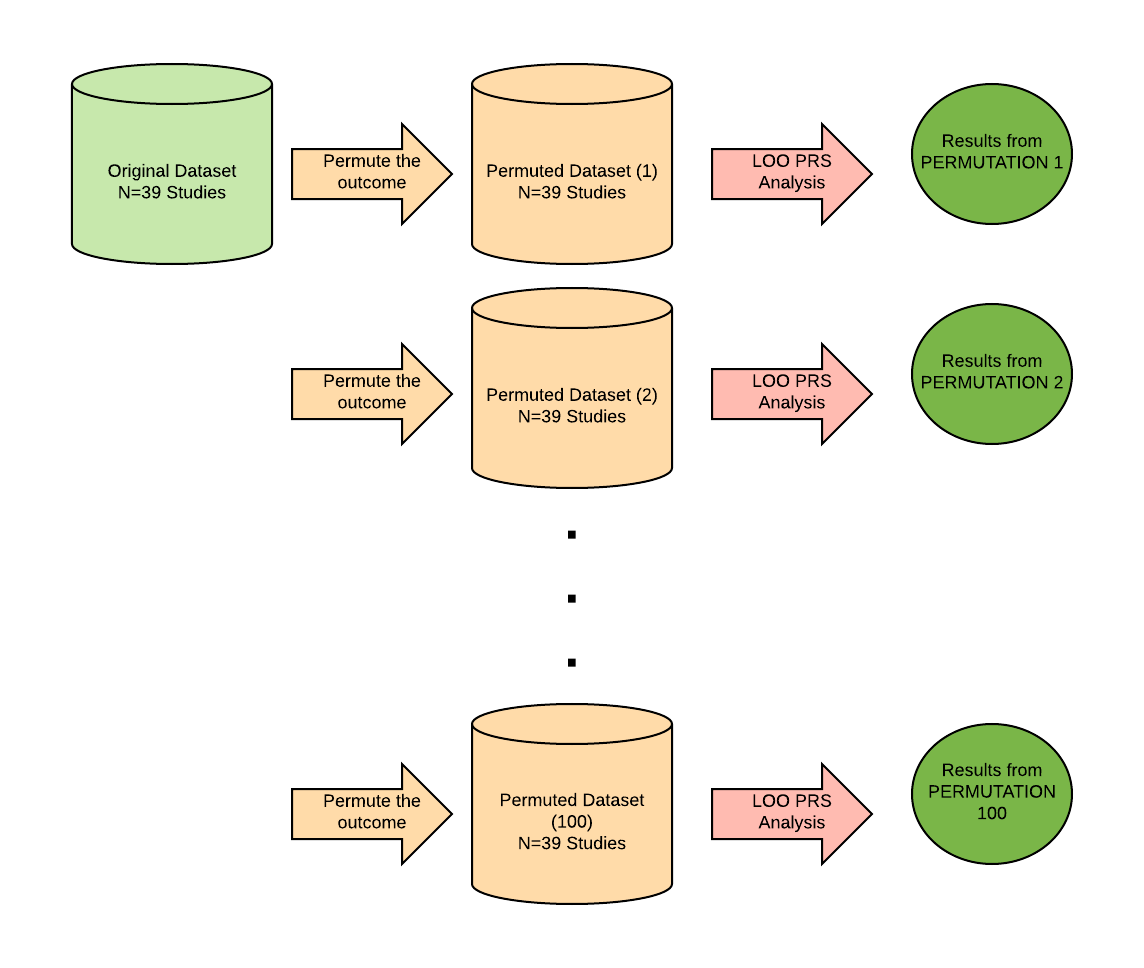


**Supplemental Figure 2: Q-Q Plot of -log_10_ P-value in the PGC2 Sample of 39 studies**


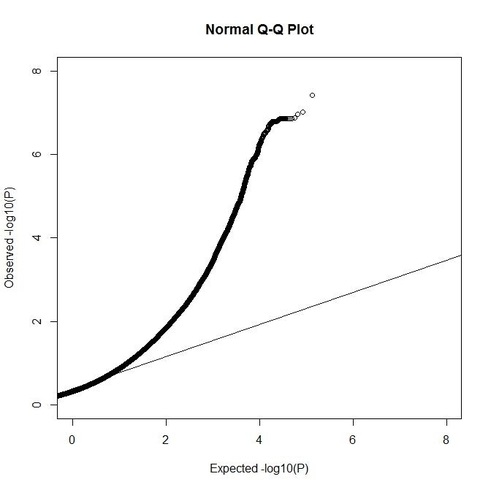


*Expected versus Observed P-values in the selected sample which included 39 studies from the PGC2 original sample.*

**Supplementary Table S1. Individual PGC Study Details**

| **Site** | **QC score** | **Array** | **Cases** | **Controls** | **Male** |
| --- | --- | --- | --- | --- | --- |
| Umeå, Sweden | 9 | omni | 341 | 577 | 0.503 |
| Umeå, Sweden | 9 | omni | 193 | 704 | 0.475 |
| Norway (TOP) | 9 | A6.0 | 377 | 403 | 0.533 |
| Edinburgh, UK | 8 | A6.0 | 367 | 284 | 0.633 |
| Seven countries (PEIC, WTCCC2) | 6 | I1M | 574 | 1812 | 0.557 |
| Spain (PEIC, WTCCC2) | 6 | I1M | 150 | 236 | 0.585 |
| New York, US & Israel | 7 | A6.0 | 325 | 139 | 0.614 |
| Ireland | 9 | A6.0 | 264 | 839 | 0.394 |
| Ireland (WTCCC2) | 9 | A6.0 | 1291 | 1006 | 0.617 |
| Germany (GRAS) | 9 | AXI | 1067 | 1169 | 0.642 |
| Estonia (EGCUT) | 2 | omni | 234 | 1152 | 0.268 |
| US, Australia (MGS) | 9 | A6.0 | 2638 | 2482 | 0.588 |
| London, UK | 8 | A6.0 | 509 | 485 | 0.572 |
| Sweden (Hubin) | 3 | omni | 265 | 319 | 0.618 |
| Bulgaria | 8 | A6.0 | 195 | 608 | 0.474 |
| Israel | 8 | I1M | 894 | 1594 | 0.701 |
| Six countries, WTCCC controls | 4 | I550 | 157 | 245 | 0.918 |
| New York, US | 8 | A500 | 190 | 190 | 0.577 |
| Australia | 9 | I650 | 456 | 287 | 0.601 |
| Cardiff, UK | 9 | A500 | 396 | 284 | 0.589 |
| UK (CLOZUK) | 0 | I1M | 3426 | 4085 | 0.88 |
| UK (CLOZUK) | 0 | omni | 2105 | 1975 | 0.629 |
| Netherlands | 7 | I550 | 700 | 607 | 0.628 |
| Portugal | 9 | A6.0 | 346 | 215 | 0.521 |
| Boston, US (CIDAR) | 9 | omni | 67 | 65 | 0.757 |
| Munich, Germany | 8 | I317 | 421 | 312 | 0.569 |
| Aberdeen, UK | 9 | A6.0 | 719 | 697 | 0.693 |
| US (CATIE) | 7 | A500 | 397 | 203 | 0.767 |
| Sweden | 3 | A5.0 | 215 | 210 | 0.527 |
| Sweden | 3 | A6.0 | 1980 | 2274 |  |
| Sweden | 3 | omni | 1764 | 2581 | 0.553 |
| Sweden | 3 | omni | 975 | 1145 | 0.543 |
| Cardiff, UK (CogUK) | 9 | omni | 530 | 678 | 0.554 |
| NIMH CBDB | 5 | O25 | 133 | 269 | 0.547 |
| NIMH CBDB | 5 | I550 | 497 | 389 | 0.627 |
| Denmark | 8 | I650 | 471 | 456 | 0.583 |
| Bulgaria (trios) | 8 | A6.0 | 649 | 649 | 0.502 |
| Six countries (trios) | 4 | I650 | 516 | 516 | 0.556 |
| Bulgaria (trios) | 8 | omni | 70 | 70 | 0.595 |

**Supplementary Table S2. Gene Ontology Enrichment**

| ***TCF4*** | **P-value** |
| --- | --- |
| biological process  cellular process  single-organism process  metabolic process  cellular component organization or biogenesis  single-organism cellular process  organic substance metabolic process  cellular metabolic process  cellular component organization  biological regulation | 4.10E-16  7.01E-15  6.32E-10  1.06E-09  2.93E-09  1.77E-08  5.30E-08  1.36E-07  2.72E-07  7.78E-07 |
| **FMRP** |  |
| nervous system development  generation of neurons  neurogenesis  neuron projection development  synaptic transmission  trans-synaptic signalling  synaptic signalling  signalling  cell communication  single organism signalling | 3.36E-60  6.97E-44  2.79E-42  2.02E-39  2.40E-36  2.40E-36  2.40E-36  6.91E-36  1.06E-35  4.63E-35 |
| **miR 137 (downregulated)** |  |
| cellular process  biological process  metabolic process  cellular metabolic process  organic substance metabolic process  primary metabolic process  cellular component organization or biogenesis  cellular component organization  single-organism process  cellular protein metabolic process | 7.51E-14  9.37E-13  1.59E-10  3.94E-10  7.84E-09  1.03E-08  1.09E-07  1.30E-07  2.37E-07  1.04E-06 |
| **miR 137 (upregulated)** |  |
| cellular process  biological process  cellular metabolic process  metabolic process  organic substance metabolic process  primary metabolic process  single-organism process  cellular macromolecule metabolic process  cellular component organization or biogenesis  single-organism cellular process | 2.23E-13  5.31E-10  2.33E-08  2.57E-07  5.43E-06  2.22E-05  4.62E-05  8.22E-05  1.55E-04  2.03E-04 |
| ***CHD8* (downregulated)** |  |
| biological process  single-organism developmental process  nervous system development  anatomical structure development  developmental process  single-multicellular organism process  multicellular organism development  system development  single-organism process  multicellular organismal process | 1.16E-08  9.85E-08  1.48E-07  1.73E-07  2.09E-07  2.14E-07  2.73E-07  1.29E-06  1.98E-06  9.68E-05 |
| ***CHD8* (upregulated)** |  |
| cellular process  biological process  cellular metabolic process  cellular component organization or biogenesis  metabolic process  primary metabolic process  cellular macromolecule metabolic process  single-organism cellular process  organic substance metabolic process  sensory perception of chemical stimulus | 5.23E-10  1.53E-09  2.55E-09  6.37E-07  1.82E-06  2.76E-06  6.37E-06  9.59E-06  9.96E-06  1.83E-05 |
| **Cancer** |  |
| positive regulation of macromolecule metabolic process  regulation of nucleobase-containing compound metabolic process  regulation of macromolecule metabolic process  regulation of nitrogen compound metabolic process  regulation of nucleic acid-templated transcription  regulation of biosynthetic process  regulation of RNA biosynthetic process  regulation of cellular biosynthetic process  positive regulation of metabolic process  regulation of metabolic process | 1.65E-81  1.99E-81  4.46E-80  4.57E-80  4.64E-80  1.33E-79  1.64E-79  2.69E-79  2.70E-79  1.56E-78 |
| **Cardiac disease** |  |
| response to chemical  response to stress  response to organic substance  response to stimulus  regulation of biological quality  response to oxygen-containing compound  response to external stimulus  regulation of multicellular organismal process  single-multicellular organism process  multicellular organismal process | 9.31E-130  6.66E-120  4.90E-117  1.97E-115  2.25E-111  1.20E-109  1.32E-107  8.07E-105  7.11E-101  4.23E-95 |

**Supplementary Table S3. Median and IQR of Genes in Gene Sets**

| GENESET | MEDIAN | IQR Lower | IQR Upper |
| --- | --- | --- | --- |
| Putative Core Gene-sets | | | |
| TCF4 | 28570 | 9332 | 68162.5 |
| FMRP | 91933 | 38483.5 | 196109.5 |
| Mir137 upregulated | 25750 | 9222.75 | 63390 |
| Mir137 downregulated | 34713 | 13899.25 | 82837 |
| CDH8 upregulated | 13827 | 6297.5 | 31674.5 |
| CDH8 downregulated | 71665 | 23439 | 167650 |
| Control Sets | | | |
| Cardiac | 23131 | 7677.5 | 71943 |
| Cancer | 52281 | 18009 | 115420 |
| Random Genic Sets | | | |
| Set1 | 55992 | 24331.5 | 120197.5 |
| Set2 | 57913 | 26497.5 | 119708 |
| Set 3 | 57192 | 26227 | 120399 |
| Set 4 | 55982 | 24929 | 118653.5 |
| Set 5 | 55999 | 25575 | 1191560 |
